# 2-Hydroxy-3-methoxybenzaldehyde 2,4-dinitrophenylhydrazone pyridine monosolvate

**DOI:** 10.1107/S1600536810029971

**Published:** 2010-08-04

**Authors:** Lin-xiu Zhao, Duan-lin Cao, Jian-lan Cui

**Affiliations:** a College of Chemical Engineering and Environment, North University of China, Taiyuan 030051, People’s Republic of China

## Abstract

The Schiff base molecule of the title compound, C_14_H_12_N_4_O_6_·C_5_H_5_N, was obtained from the condensation reaction of 2-hy­droxy-3-meth­oxy­benzaldehyde and 2,4-dinitro­phenyl­hydrazine. The C=N bond of the Schiff base has a *trans* arrangement and the dihedral angle between the two benzene rings is 3.49 (10)°. An intra­molecular N—H⋯O hydrogen bond generates an *S*(6) ring. In the crystal, O—H⋯O hydrogen bonds link the Schiff base mol­ecules.

## Related literature

For background to Schiff bases, see: Kahwa *et al.* (1986 ▶); Santos *et al.* (2001 ▶). For a related structure, see: Ohba (1996 ▶).
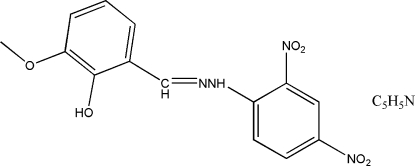


## Experimental

### 

#### Crystal data

C_14_H_12_N_4_O_6_·C_5_H_5_N
*M* *_r_* = 411.38
Triclinic, 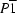

*a* = 6.9020 (18) Å
*b* = 7.6240 (12) Å
*c* = 19.073 (3) Å
α = 95.112 (13)°
β = 91.199 (17)°
γ = 107.024 (19)°
*V* = 954.7 (3) Å^3^
*Z* = 2
Mo *K*α radiation
μ = 0.11 mm^−1^
*T* = 293 K
0.21 × 0.19 × 0.17 mm

#### Data collection

Bruker SMART CCD diffractometer
Absorption correction: multi-scan (*SADABS*; Bruker, 1998 ▶) *T*_min_ = 0.973, *T*_max_ = 0.978
7517 measured reflections
4401 independent reflections
1852 reflections with *I* > 2σ(*I*)
*R*_int_ = 0.020

#### Refinement

*R*[*F* ^2^ > 2σ(*F* ^2^)] = 0.051
*wR*(*F* ^2^) = 0.143
*S* = 0.82
4401 reflections
271 parameters
H-atom parameters constrained
Δρ_max_ = 0.31 e Å^−3^
Δρ_min_ = −0.38 e Å^−3^

### 

Data collection: *SMART* (Bruker, 1998 ▶); cell refinement: *SAINT* (Bruker, 1998 ▶); data reduction: *SAINT*; program(s) used to solve structure: *SHELXTL* (Sheldrick, 2008 ▶); program(s) used to refine structure: *SHELXTL*; molecular graphics: *SHELXTL*; software used to prepare material for publication: *SHELXTL*.

## Supplementary Material

Crystal structure: contains datablocks global, I. DOI: 10.1107/S1600536810029971/hb5577sup1.cif

Structure factors: contains datablocks I. DOI: 10.1107/S1600536810029971/hb5577Isup2.hkl

Additional supplementary materials:  crystallographic information; 3D view; checkCIF report

## Figures and Tables

**Table 1:** Hydrogen-bond geometry (Å, °)

*D*—H⋯*A*	*D*—H	H⋯*A*	*D*⋯*A*	*D*—H⋯*A*
O1—H1*B*⋯O4^i^	0.82	2.53	3.319 (2)	162
N2—H2*A*⋯O3	0.86	2.03	2.635 (2)	126

Symmetry code: (i) 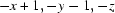.

**Fig. 1.**
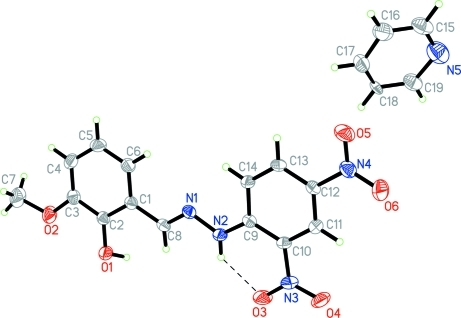
: The molecular structure of (I) with displacement ellipsoids drawn at the 30% probability level. The intramolecular hydrogen bond is indicatd with dashed lines.

**Fig. 2.**
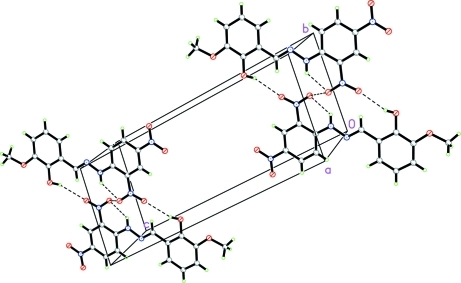
The partial packing of (I).

## supplementary crystallographic information

### Comment

The chemistry of Schiff base has attracted a great deal of interest in recent years. These compounds play an important role in the development of various proteins and enzymes (Kahwa *et al.*, 1986; Santos *et al.*, 2001). As part of our in the study of the coordination chemistry of Schiff bases, we synthesized the title compound, (I), and determined its crystal structure.

The molecular structure of (I) is shown in Fig.1. The benzene ring and the 2,4-dinitro benzene ring are nearly coplanar, making a dihedral angle of 3.49 (10)°. The dinitro group is coplanar with C9-benzene ring with a dihedral angle of 0.58 (8). Bond lengths and bond angles agree with those of other dinitrophenylhydrazone derivatives (Ohba, 1996).

Intramolecular N—H···O and intermolecular O—H···O hydrogen bonds are certainly responsible for the planar conformation of the molecule.

### Experimental

2,4-Dinitrophenylhydrazine (1 mmol, 0.198 g) was dissolved in anhydrous ethanol (15 ml), H_2_SO_4_(98%, 0.5 ml) was then added and the mixture was stirred for several minitutes at 351 K. Then, 2-hydroxy-3-methoxybenzaldehyde (1 mmol, 0.152 g) in ethanol (8 mm l) was added dropwise and the mixture was stirred at refluxing temperature for 3 h. The product was isolated and recrystallized from pyridine, red blocks of (I) were obtained after two weeks.

### Refinement

All H atoms were positioned geometrically and refined as riding with C—H = 0.93 (aromatic), 0.96 Å(methyl) and N—H = 0.86 Å, with *U*_iso_(H) = 1.2*U*_eq_(CH, CH2 or NH) and *U*_iso_(H) = 1.5*U*_eq_(C).

### Crystal data

**Table:**

C_14_H_12_N_4_O_6_·C_5_H_5_N	*Z* = 2
*M**_r_* = 411.38	*F*(000) = 418
Triclinic, *P*1	*D*_x_ = 1.431 Mg m^−^^3^
Hall symbol: -P 1	Mo *K*α radiation, λ = 0.71073 Å
*a* = 6.9020 (18) Å	Cell parameters from 1741 reflections
*b* = 7.6240 (12) Å	θ = 3.2–29.3°
*c* = 19.073 (3) Å	µ = 0.11 mm^−^^1^
α = 95.112 (13)°	*T* = 293 K
β = 91.199 (17)°	Block, red
γ = 107.024 (19)°	0.21 × 0.19 × 0.17 mm
*V* = 954.7 (3) Å^3^	

### Data collection

**Table:**

Bruker SMART CCD diffractometer	4401 independent reflections
Radiation source: fine-focus sealed tube	1852 reflections with *I* > 2σ(*I*)
graphite	*R*_int_ = 0.020
ω scans	θ_max_ = 29.3°, θ_min_ = 3.2°
Absorption correction: multi-scan (*SADABS*; Bruker, 1998)	*h* = −8→9
*T*_min_ = 0.973, *T*_max_ = 0.978	*k* = −10→8
7517 measured reflections	*l* = −23→25

### Refinement

**Table:**

Refinement on *F*^2^	Primary atom site location: structure-invariant direct methods
Least-squares matrix: full	Secondary atom site location: difference Fourier map
*R*[*F*^2^ > 2σ(*F*^2^)] = 0.051	Hydrogen site location: inferred from neighbouring sites
*wR*(*F*^2^) = 0.143	H-atom parameters constrained
*S* = 0.82	*w* = 1/[σ^2^(*F*_o_^2^) + (0.078*P*)^2^] where *P* = (*F*_o_^2^ + 2*F*_c_^2^)/3
4401 reflections	(Δ/σ)_max_ < 0.001
271 parameters	Δρ_max_ = 0.31 e Å^−^^3^
0 restraints	Δρ_min_ = −0.38 e Å^−^^3^

### Special details

**Table:**

Geometry. All e.s.d.'s (except the e.s.d. in the dihedral angle between two l.s. planes) are estimated using the full covariance matrix. The cell e.s.d.'s are taken into account individually in the estimation of e.s.d.'s in distances, angles and torsion angles; correlations between e.s.d.'s in cell parameters are only used when they are defined by crystal symmetry. An approximate (isotropic) treatment of cell e.s.d.'s is used for estimating e.s.d.'s involving l.s. planes.
Refinement. Refinement of *F*^2^ against ALL reflections. The weighted *R*-factor *wR* and goodness of fit *S* are based on *F*^2^, conventional *R*-factors *R* are based on *F*, with *F* set to zero for negative *F*^2^. The threshold expression of *F*^2^ > σ(*F*^2^) is used only for calculating *R*-factors(gt) *etc*. and is not relevant to the choice of reflections for refinement. *R*-factors based on *F*^2^ are statistically about twice as large as those based on *F*, and *R*- factors based on ALL data will be even larger.

### Fractional atomic coordinates and isotropic or equivalent isotropic displacement parameters (Å^2^)

**Table:**

	*x*	*y*	*z*	*U*_iso_*/*U*_eq_	
N1	0.7466 (3)	0.0112 (2)	0.01896 (10)	0.0424 (5)	
N3	0.6500 (3)	−0.4948 (2)	−0.11703 (12)	0.0485 (5)	
O1	0.7209 (2)	0.00916 (18)	0.23110 (8)	0.0549 (5)	
H1B	0.6315	−0.0690	0.2069	0.082*	
C1	0.7623 (3)	0.1767 (3)	0.13134 (12)	0.0390 (5)	
N2	0.7090 (3)	−0.1535 (2)	−0.02176 (10)	0.0429 (5)	
H2A	0.6742	−0.2561	−0.0030	0.051*	
C2	0.7567 (3)	0.1720 (3)	0.20412 (13)	0.0412 (6)	
O3	0.6370 (3)	−0.5111 (2)	−0.05341 (11)	0.0677 (5)	
C12	0.7671 (3)	−0.1328 (3)	−0.23637 (12)	0.0386 (5)	
O2	0.7845 (3)	0.3159 (2)	0.31832 (9)	0.0670 (5)	
C8	0.7250 (3)	0.0051 (3)	0.08483 (13)	0.0399 (5)	
H8A	0.6856	−0.1080	0.1035	0.048*	
C10	0.7001 (3)	−0.3115 (2)	−0.13946 (12)	0.0367 (5)	
C3	0.7921 (3)	0.3370 (3)	0.24820 (13)	0.0476 (6)	
C6	0.8044 (3)	0.3474 (3)	0.10356 (13)	0.0477 (6)	
H6A	0.8102	0.3526	0.0551	0.057*	
C14	0.7760 (3)	0.0185 (3)	−0.12116 (12)	0.0400 (5)	
H14A	0.7952	0.1268	−0.0918	0.048*	
C11	0.7186 (3)	−0.3009 (3)	−0.21097 (12)	0.0399 (6)	
H11A	0.6983	−0.4075	−0.2415	0.048*	
O4	0.6221 (3)	−0.6271 (2)	−0.16096 (10)	0.0679 (6)	
N4	0.7912 (3)	−0.1221 (3)	−0.31121 (11)	0.0534 (5)	
C5	0.8368 (3)	0.5061 (3)	0.14741 (15)	0.0549 (7)	
H5A	0.8634	0.6184	0.1284	0.066*	
C13	0.7946 (3)	0.0271 (3)	−0.19136 (12)	0.0407 (6)	
H13A	0.8261	0.1407	−0.2096	0.049*	
O5	0.8451 (3)	0.0308 (2)	−0.33278 (10)	0.0713 (6)	
C9	0.7280 (3)	−0.1511 (3)	−0.09170 (12)	0.0354 (5)	
C4	0.8307 (3)	0.5021 (3)	0.21998 (15)	0.0533 (7)	
H4A	0.8528	0.6111	0.2493	0.064*	
O6	0.7599 (3)	−0.2658 (3)	−0.35043 (10)	0.0813 (6)	
C18	0.3624 (4)	0.0818 (3)	−0.36369 (12)	0.0432 (6)	
H18A	0.3344	0.0297	−0.3214	0.052*	
C7	0.8415 (5)	0.4749 (4)	0.36657 (16)	0.0861 (10)	
H7A	0.8292	0.4398	0.4138	0.129*	
H7B	0.7546	0.5500	0.3588	0.129*	
H7C	0.9796	0.5435	0.3601	0.129*	
N5	0.2789 (4)	0.0847 (4)	−0.48479 (16)	0.0962 (9)	
C19	0.2479 (4)	0.0124 (4)	−0.42066 (17)	0.0663 (8)	
H19A	0.1386	−0.0923	−0.4179	0.080*	
C17	0.5183 (5)	0.2274 (4)	−0.36801 (16)	0.0732 (9)	
H17A	0.6012	0.2766	−0.3277	0.088*	
C15	0.4455 (5)	0.2404 (4)	−0.48748 (17)	0.0736 (8)	
H15A	0.4736	0.2948	−0.5293	0.088*	
C16	0.5660 (5)	0.3114 (4)	−0.42861 (18)	0.0760 (9)	
H16A	0.6783	0.4146	−0.4293	0.091*	

### Atomic displacement parameters (Å^2^)

**Table:**

	*U*^11^	*U*^22^	*U*^33^	*U*^12^	*U*^13^	*U*^23^
N1	0.0478 (11)	0.0423 (10)	0.0358 (13)	0.0098 (8)	0.0002 (9)	0.0099 (9)
N3	0.0542 (13)	0.0366 (11)	0.0584 (16)	0.0161 (9)	0.0098 (11)	0.0130 (11)
O1	0.0749 (11)	0.0411 (9)	0.0432 (11)	0.0083 (7)	−0.0017 (9)	0.0069 (7)
C1	0.0306 (11)	0.0417 (12)	0.0435 (16)	0.0081 (9)	−0.0015 (10)	0.0074 (10)
N2	0.0563 (12)	0.0342 (9)	0.0379 (12)	0.0107 (8)	0.0036 (10)	0.0109 (8)
C2	0.0362 (12)	0.0440 (12)	0.0425 (16)	0.0099 (9)	−0.0003 (11)	0.0060 (11)
O3	0.1054 (15)	0.0491 (10)	0.0544 (14)	0.0253 (9)	0.0186 (11)	0.0247 (9)
C12	0.0393 (12)	0.0444 (12)	0.0343 (14)	0.0142 (9)	0.0037 (10)	0.0097 (10)
O2	0.0895 (13)	0.0634 (10)	0.0400 (12)	0.0122 (9)	0.0021 (10)	−0.0021 (9)
C8	0.0380 (13)	0.0438 (12)	0.0390 (15)	0.0116 (9)	−0.0002 (11)	0.0123 (10)
C10	0.0346 (12)	0.0317 (11)	0.0448 (15)	0.0093 (9)	0.0043 (10)	0.0104 (10)
C3	0.0421 (13)	0.0523 (14)	0.0452 (17)	0.0094 (10)	0.0008 (12)	0.0036 (12)
C6	0.0434 (13)	0.0469 (13)	0.0480 (16)	0.0039 (10)	−0.0011 (12)	0.0123 (11)
C14	0.0472 (13)	0.0343 (11)	0.0377 (15)	0.0100 (9)	0.0037 (11)	0.0070 (10)
C11	0.0398 (13)	0.0358 (11)	0.0430 (16)	0.0102 (9)	0.0029 (11)	0.0016 (10)
O4	0.0970 (14)	0.0336 (9)	0.0729 (14)	0.0187 (9)	0.0124 (11)	0.0037 (9)
N4	0.0635 (14)	0.0614 (13)	0.0397 (14)	0.0235 (11)	0.0074 (10)	0.0110 (11)
C5	0.0510 (15)	0.0418 (13)	0.069 (2)	0.0058 (11)	0.0002 (13)	0.0178 (12)
C13	0.0457 (13)	0.0338 (11)	0.0427 (15)	0.0083 (9)	0.0071 (11)	0.0150 (10)
O5	0.1020 (15)	0.0721 (12)	0.0476 (13)	0.0311 (10)	0.0184 (10)	0.0256 (9)
C9	0.0302 (11)	0.0384 (11)	0.0376 (15)	0.0082 (9)	0.0039 (10)	0.0101 (10)
C4	0.0508 (15)	0.0434 (13)	0.060 (2)	0.0067 (11)	−0.0025 (13)	−0.0023 (12)
O6	0.1247 (17)	0.0754 (12)	0.0429 (12)	0.0308 (11)	0.0063 (11)	−0.0032 (10)
C18	0.0617 (15)	0.0504 (13)	0.0200 (13)	0.0172 (12)	0.0036 (11)	0.0137 (10)
C7	0.115 (3)	0.084 (2)	0.053 (2)	0.0256 (18)	0.0025 (18)	−0.0192 (16)
N5	0.102 (2)	0.120 (2)	0.080 (2)	0.0501 (18)	0.0147 (17)	0.0221 (18)
C19	0.0695 (19)	0.0749 (17)	0.058 (2)	0.0208 (14)	0.0169 (16)	0.0237 (15)
C17	0.099 (2)	0.0744 (19)	0.054 (2)	0.0404 (17)	−0.0206 (18)	0.0007 (16)
C15	0.084 (2)	0.0792 (19)	0.067 (2)	0.0295 (17)	0.0219 (18)	0.0330 (17)
C16	0.081 (2)	0.0683 (17)	0.079 (3)	0.0186 (15)	−0.0033 (19)	0.0186 (17)

### Geometric parameters (Å, °)

**Table:**

N1—C8	1.272 (3)	C14—C13	1.353 (3)
N1—N2	1.369 (2)	C14—C9	1.408 (3)
N3—O4	1.219 (2)	C14—H14A	0.9300
N3—O3	1.233 (2)	C11—H11A	0.9300
N3—C10	1.444 (3)	N4—O5	1.227 (2)
O1—C2	1.344 (2)	N4—O6	1.231 (2)
O1—H1B	0.8200	C5—C4	1.388 (4)
C1—C2	1.393 (3)	C5—H5A	0.9300
C1—C6	1.402 (3)	C13—H13A	0.9300
C1—C8	1.467 (3)	C4—H4A	0.9300
N2—C9	1.344 (3)	C18—C19	1.305 (3)
N2—H2A	0.8600	C18—C17	1.310 (3)
C2—C3	1.405 (3)	C18—H18A	0.9300
C12—C11	1.362 (3)	C7—H7A	0.9600
C12—C13	1.389 (3)	C7—H7B	0.9600
C12—N4	1.447 (3)	C7—H7C	0.9600
O2—C3	1.362 (3)	N5—C19	1.381 (4)
O2—C7	1.407 (3)	N5—C15	1.396 (4)
C8—H8A	0.9300	C19—H19A	0.9300
C10—C11	1.380 (3)	C17—C16	1.371 (4)
C10—C9	1.421 (3)	C17—H17A	0.9300
C3—C4	1.370 (3)	C15—C16	1.354 (4)
C6—C5	1.366 (3)	C15—H15A	0.9300
C6—H6A	0.9300	C16—H16A	0.9300
			
C8—N1—N2	117.18 (18)	O5—N4—C12	118.34 (19)
O4—N3—O3	122.35 (19)	O6—N4—C12	119.0 (2)
O4—N3—C10	119.5 (2)	C6—C5—C4	121.0 (2)
O3—N3—C10	118.15 (19)	C6—C5—H5A	119.5
C2—O1—H1B	109.5	C4—C5—H5A	119.5
C2—C1—C6	119.0 (2)	C14—C13—C12	120.38 (19)
C2—C1—C8	120.1 (2)	C14—C13—H13A	119.8
C6—C1—C8	120.8 (2)	C12—C13—H13A	119.8
C9—N2—N1	118.40 (17)	N2—C9—C14	119.45 (18)
C9—N2—H2A	120.8	N2—C9—C10	124.01 (19)
N1—N2—H2A	120.8	C14—C9—C10	116.5 (2)
O1—C2—C1	119.27 (19)	C3—C4—C5	119.7 (2)
O1—C2—C3	121.0 (2)	C3—C4—H4A	120.2
C1—C2—C3	119.7 (2)	C5—C4—H4A	120.2
C11—C12—C13	120.8 (2)	C19—C18—C17	117.8 (3)
C11—C12—N4	119.12 (19)	C19—C18—H18A	121.1
C13—C12—N4	120.05 (19)	C17—C18—H18A	121.1
C3—O2—C7	118.4 (2)	O2—C7—H7A	109.5
N1—C8—C1	119.9 (2)	O2—C7—H7B	109.5
N1—C8—H8A	120.1	H7A—C7—H7B	109.5
C1—C8—H8A	120.1	O2—C7—H7C	109.5
C11—C10—C9	121.50 (19)	H7A—C7—H7C	109.5
C11—C10—N3	115.69 (19)	H7B—C7—H7C	109.5
C9—C10—N3	122.8 (2)	C19—N5—C15	116.6 (3)
O2—C3—C4	125.1 (2)	C18—C19—N5	124.1 (3)
O2—C3—C2	114.6 (2)	C18—C19—H19A	118.0
C4—C3—C2	120.4 (2)	N5—C19—H19A	118.0
C5—C6—C1	120.3 (2)	C18—C17—C16	123.7 (3)
C5—C6—H6A	119.9	C18—C17—H17A	118.1
C1—C6—H6A	119.9	C16—C17—H17A	118.1
C13—C14—C9	121.41 (19)	C16—C15—N5	119.4 (3)
C13—C14—H14A	119.3	C16—C15—H15A	120.3
C9—C14—H14A	119.3	N5—C15—H15A	120.3
C12—C11—C10	119.34 (19)	C15—C16—C17	118.3 (3)
C12—C11—H11A	120.3	C15—C16—H16A	120.8
C10—C11—H11A	120.3	C17—C16—H16A	120.8
O5—N4—O6	122.7 (2)		

### Hydrogen-bond geometry (Å, °)

**Table:**

*D*—H···*A*	*D*—H	H···*A*	*D*···*A*	*D*—H···*A*
O1—H1B···O4^i^	0.82	2.53	3.319 (2)	162.
N2—H2A···O3	0.86	2.03	2.635 (2)	126.

Symmetry codes: (i) −*x*+1, −*y*−1, −*z*.
